# (−)-Loliolide Isolated from *Sargassum horneri* Abate UVB-Induced Oxidative Damage in Human Dermal Fibroblasts and Subside ECM Degradation

**DOI:** 10.3390/md19080435

**Published:** 2021-07-30

**Authors:** Ilekuttige Priyan Shanura Fernando, Soo-Jin Heo, Mawalle Kankanamge Hasitha Madhawa Dias, Dissanayaka Mudiyanselage Dinesh Madusanka, Eui-Jeong Han, Min-Ju Kim, Kalu Kapuge Asanka Sanjeewa, Kyounghoon Lee, Ginnae Ahn

**Affiliations:** 1 Department of Marine Bio-Food Sciences, Chonnam National University, Yeosu 59626, Korea; shanura@chonnam.ac.kr; 2 Jeju Marine Research Center, Korea Institute of Ocean Science & Technology (KIOST), Jeju 63349, Korea; sjheo@kiost.ac.kr; 3 Department of Food Technology and Nutrition, Chonnam National University, Yeosu 59626, Korea; 198807@jnu.ac.kr (M.K.H.M.D.); 198793@jnu.ac.kr (D.M.D.M.); iosu5772@jnu.ac.kr (E.-J.H.); 177537@jnu.ac.kr (M.-J.K.); 4 Department of Biosystems Technology, Faculty of Technology, University of Sri Jayewardenepura, Sri Jayewardenepura 10206, Sri Lanka; asanka@jejunu.ac.kr; 5 Division of Fisheries Science, Chonnam National University, Yeosu 59626, Korea; 6 Department of Marine Technology, Chonnam National University, Yeosu 59626, Korea

**Keywords:** ultraviolet B, (−)-loliolide, *Sargassum horneri*, dermal fibroblasts, matrix metalloproteinase

## Abstract

Ultraviolet (UV) B exposure is a prominent cause of skin aging and a contemporary subject of interest. The effects are progressing through the generation of reactive oxygen species (ROS) that alter cell signaling pathways related to inflammatory responses. The present study evaluates the protective effects of (7aR)-6-hydroxy-4,4,7a-trimethyl-6,7-dihydro-5H-1-benzofuran-2-one (HTT) isolated from the edible brown algae Sargassum horneri against UVB protective effects in human dermal fibroblasts (HDFs). HTT treatment dose-dependently suppressed intracellular ROS generation in HDFs with an IC_50_ of 62.43 ± 3.22 µM. HTT abated UVB-induced mitochondrial hyperpolarization and apoptotic body formation. Furthermore, UVB-induced activation of key nuclear factor (NF)-κB and mitogen-activated protein kinase signaling proteins were suppressed in HTT treated cells while downregulating pro-inflammatory cytokines (interleukin-1β, 6, 8, 33 and tumor necrosis factor-α). Moreover, HTT treatment downregulated matrix metalloproteinase1, 2, 3, 8, 9 and 13 that was further confirmed by the inhibition of collagenase and elastase activity. The evidence implies that HTT delivers protective effects against premature skin aging caused by UVB exposure via suppressing inflammatory responses and degradation of extracellular matrix (ECM) components. Extensive research in this regard will raise perspectives for using HTT as an ingredient in UV protective ointments.

## 1. Introduction

Premature skin aging is a condition characterized by hyperpigmentation, wrinkling, loss of hair, and thickening that results in flaky, dry, and itchy skin [1]. These symptoms can arise due to intrinsic factors such as genetics, hormonal imbalances, weight loss, and dysregulated cell signaling as well as extrinsic factors including chronic exposure to UV and other forms of ionizing radiation, environmental conditions, pathogenic microorganisms, hazardous chemicals, and lifestyle patterns. 

Sunlight which is comprised of a continuous spectrum of electromagnetic radiation is a source of ultraviolet (UV 200–400 nm) radiation [2]. In the majority of people, ultraviolet (UV) radiation is the main environmental factor responsible for skin aging [3]. Photoaged skin typically indicates a thickened epidermis, deep wrinkles, loss of elasticity, laxity, mottled discoloration, dullness, and roughness with a reduced epidermal turnover rate [1]. Based on the wavelength, UV radiation is classified into UVA (400–320 nm), UVB (320–280 nm), and UVC (280–100 nm). Among them, UVA and UVB can penetrate through the dermis affecting the underlying keratinocytes and fibroblasts. Studies have demonstrated that photodamage causes the accumulation of lipid peroxides and glycation end products, and downregulates antioxidant enzymes while increasing posttranslational protein modifications [4,5]. 

Dermal fibroblasts, which secrete collagen, elastin, and other fibrous connective tissues, are largely responsible for the skin’s structural integrity. Collagen is mainly accountable for providing tensile strength while elastin is responsible for maintaining elasticity [6]. UV radiation promotes cellular oxidative stress and inflammatory processes, resulting in a cascade of reactions that lead to connective tissue degradation in the extracellular matrix (ECM) [7]. Enzymes involved in the degradation of connective tissues include matrix metalloproteinases (MMPs). These endopeptidases degrade macromolecular components in the ECM and the basement membrane [8]. Human cells are capable of synthesizing at least 16 types of MMPs, which can be grouped as collagenases, gelatinases, stromelysins, and membrane MMPs. MMP1, MMP8, and MMP13 are three distinct collagenases expressed by human dermal fibroblasts under specific circumstances, including oxidative stress and inflammatory processes [9]. The synthesis of MMP1, MMP2, MMP3, MMP7, MMP8, MMP9, and MMP12 have witnessed an upregulation upon UV exposure of human or mouse skin, which is implicated in photoaging. 

Sunscreen formulations are designed to protect skin from UV radiation by either creating a protective barrier on the skin surface or absorbing the damaging rays. These formulations include ingredients such as titanium dioxide, zinc oxide, oxybenzone, octacrylene, avobenzone, etc. [2]. However, none of the substances listed above have antioxidant properties that protect the skin tissues from free radical damage caused by UV radiation. Recently, natural products of marine algae have gained considerable interest for their wide-ranging bioactive potential. The invasive algae, *Sargassum horneri* is recently acknowledged as a reservoir of bioactive metabolites possessing antioxidant and anti-inflammatory functionalities [10]. Among them is (−)-loliolide, which has shown desirable effects against intracellular oxidative stress generated by various factors such as 2,2’-Azobis(2-amidinopropane) dihydrochloride (AAPH), and fine dust, anti-inflammatory effects against LPS, and lipid accumulation inhibitory effects in differentiated adipocytes [11,12,13]. Considering the closely related pathophysiological processes, oxidative stress, inflammation, and MMP expression, we hypothesized that (−)-loliolide would suppress inflammatory responses and levels of MMPs implicated in UVB-induced skin photoaging.

## 2. Results

### 2.1. HTT Attenuated Intracellular ROS Level While Ameliorating HDFs Viability

**Figure 1:**
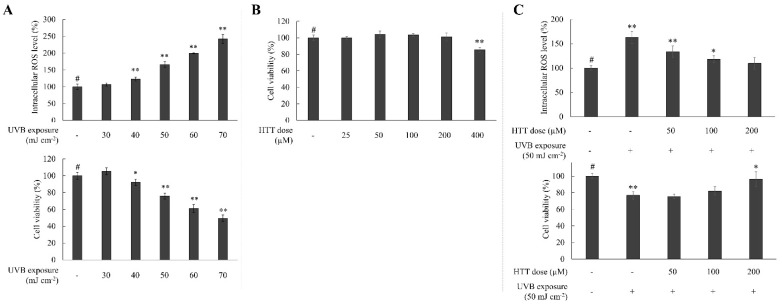
Optimization of UVB exposure, dose-response cytotoxicity, and UVB protective responses of HTT on HDFs. Evaluation of (**A**) intracellular ROS level and cell viability upon exposure of HDFs to different UVB doses. (**B**) Dose-response cytotoxicity of HTT on HDFs after a 24 h incubation period. (**C**) Protective effects of non-cytotoxic HTT doses on intracellular ROS level and HDF viability. Intracellular ROS level was measured 2 h after the stimulation or treatment whereas cell viability was measured after 24 h. Results represent the mean ± SD (error bars) of three independent experimental trials (*n* = 3). *p*-values less than 0.05 “*” and 0.001 “**” were considered as significant compared to the control group “#”.

Optimization of UVB dose indicated that the 50 mJ cm^−2^ dose, which indicated cell viability of 75.88%, is desirable for further evaluations (Figure 1A) due to the significantly high ROS level and its possibility of recovering. Herein, the intracellular ROS level indicated a dose-dependent increase with the UVB dose. At the UVB dose of 50 mJ cm^−2^, HDFs indicated an intracellular ROS level of 166.11%. UVB exposure of 60 mJ cm^−2^ was not selected as a cell viability of 61.02% was found to be practically un-recoverable with sample treatment (data not shown). HTT doses 50–200 µM was selected desirable for further analysis due to their nontoxic nature (Figure 1B). As seen in Figure 1C, UVB exposure caused a significant increase in intracellular ROS level and a decrease in HDFs viability. However, HTT treated cells had a protective response against UVB exposure, whereas it decreased the intracellular ROS level while increasing cell viability. To further confirm the ROS inhibitory effects, HDFs were analyzed by fluorescence microscopy and flow cytometry (Supplementary Materials Figure S1). Per analysis, the green fluorescence intensity indicated a prompt increase in UVB exposed HDFs and indicated a dose-dependent reduction in HTT treated cells. Collectively, these results indicate the antioxidant potential of HTT in UVB-exposed HDFs.

### 2.2. HTT Abated UVB-Induced Mitochondrial Hyperpolarization and Apoptotic Body Formation

**Figure 2:**
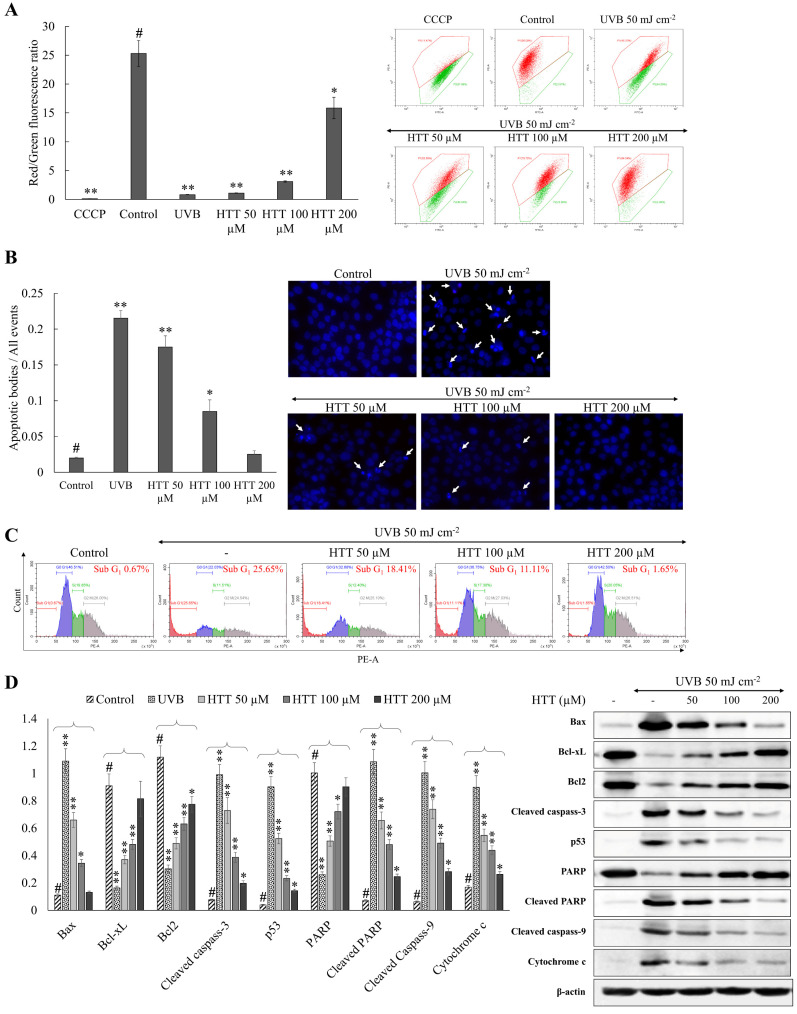
Effects of HTT in reducing UVB-induced mitochondrial damage and apoptosis in HDFs. Evaluation of (**A**) mitochondrial membrane hyperpolarization upon exposure to UVB. Evaluating apoptosis via (**B**) apoptotic body formation and (**C**) population of sub-G_1_ hypodiploid cells per cell cycle analysis. (**D**) Role of HTT in regulating mitochondria-mediated apoptotic proteins in UVB-induced keratinocytes. JC-1 assay was carried out 4 h after the UVB exposure while Hoechst 33342 nuclear staining, cell-cycle analysis, and western blotting were performed 24 h after the stimulation. Results represent the mean ± SD (error bars) of three independent experimental trials (*n* = 3). *p*-values less than 0.05 “*” and 0.001 “**” were considered as significant compared to the control group “#”.

Oxidative stress resulting from UVB distorts the permeability of the mitochondrial membrane with the subsequent outflow of factors that initiate apoptosis [14]. According to Figure 2A, UVB-treated cells indicated a decreased red-green fluorescence ratio indicative of mitochondrial hyperpolarization as compared to the control group. Recovery from this effect was seen in HTT treated cells. According to the results of Hoechst 33342 nuclear staining, UVB exposed cells indicated a higher prevalence of nuclear condensation and fragmentation compared to the control (Figure 2B). The represented figure corresponds to an area in the culture dish where a higher proportion of apoptotic bodies were observed. HTT treatment reduced the occurrence of apoptotic bodies further agreeing with the reduction seen in mitochondrial damage. Cell-cycle analysis of UVB exposed cells (Figure 2C) indicated a higher population of apoptotic hypodiploid cells residing in the sub-G_1_ stage (25.65%). In the control group, the sub-G_1_ population was 0.67%. Cells treated with HTT indicated a subsequent reduction of sub-G_1_ cell populations agreeing with inhibition of apoptosis. The events were under the regulation of the mitochondria-mediated apoptosis pathway (Figure 2D). HTT treatment significantly and dose-dependently increased the production of anti-apoptotic proteins Bcl-2 and Bcl-xL and reduced the pro-apoptotic proteins Bax that was disoriented due to UVB exposure. Moreover, a marked decrease of cytochrome c levels and cleavage of caspases 3 and 9 with reduction of PARP cleavage suggest the protective effects of HTT against UVB-induced apoptosis in HDFs. The apoptosis marker and transcription factor p53 that regulate nearly 500 target genes involved in cell senescence, cell cycle arrest, metabolic adaptation, DNA repair, and cell death indicated a dose-dependent reduction with HTT treatment supporting UVB protective effects of HTT. 

### 2.3. HTT Ameliorated UVB-Induced Irregularities in MAPK and NF-κB Signaling

**Figure 3:**
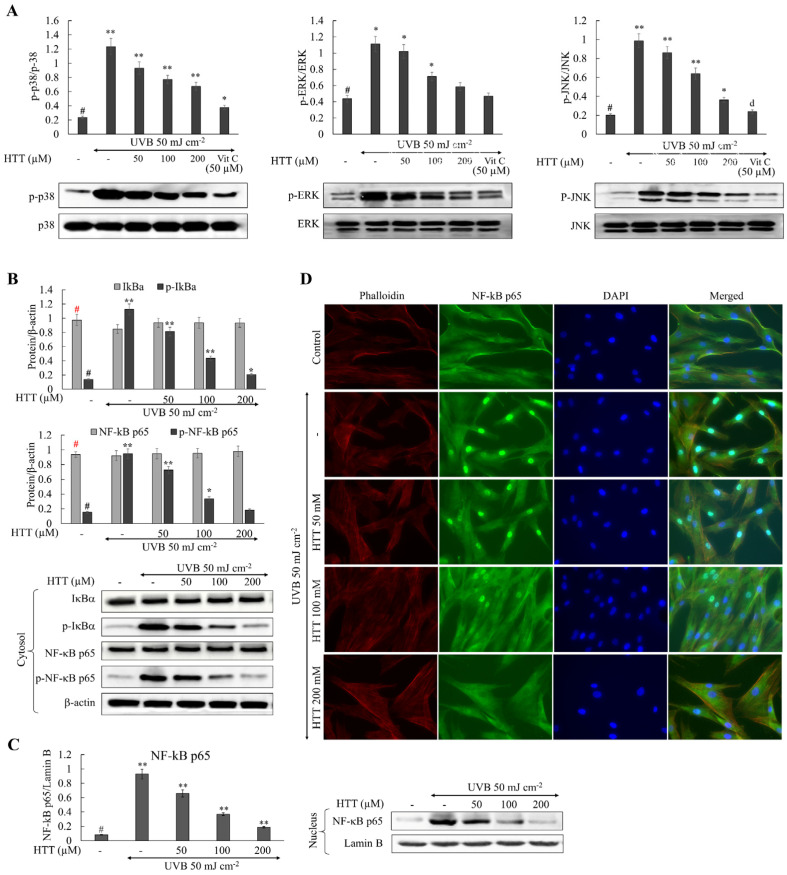
HTT reduces the phosphorylation of key NF-κB mediators and nuclear translocation of transcription factor NF-κB p65 in UVB-induced HDFs. (**A**) Suppressive effects of HTT towards UVB-induced phosphorylation of MAPK mediators. Evaluating the potential of HTT towards the suppression of UVB-induced (**B**) phosphorylation of key NF-κB mediators and (**C**) nuclear translocation of transcription factor NF-κB p65. (**D**) Immunofluorescence analysis of NF-κB p65 nuclear translocation. HDFs were either harvested or fixed 30 min after the UVB stimulation respectively for western blot and immunofluorescence analysis. Results represent the mean ± SD (error bars) of three independent experimental trials (*n* = 3). *p*-values less than 0.05 “*” and 0.001 “**” were considered as significant compared to the control group “#”.

Substantial arguments exist that ROS mediate the activation of the nuclear factor kappa-light-chain-enhancer of activated B cells (NF-κB) [15]. ROS generation by mitochondria due to Bcl-xL sequestration causes the unbinding of apoptosis signal-regulating kinase-1 (ASK-1) from thioredoxin that activates its downstream targets mitogen-activated protein kinase (MAPK) p38 and c-Jun *N*-terminal kinases (JNK) [16]. IκBα and p65, and MAPK proteins including, p38, ERK, and JNK compared to the control. In HTT pretreated cells the phosphorylation of the aforementioned molecular mediators indicated a dose-dependent reduction elaborating its potential towards suppressing upstream mediators of inflammatory responses. Immunofluorescence analysis of the transcription factor NF-κB p65 nuclear translocation further confirmed the aforementioned scenario where HTT dose-dependently suppressed the UVB induced upregulation of NF-κB p65 nuclear translocation (Figure 3C).

### 2.4. HTT Suppressed UVB-Induced Expression of Inflammatory Cytokines in HDFs

**Figure 4:**
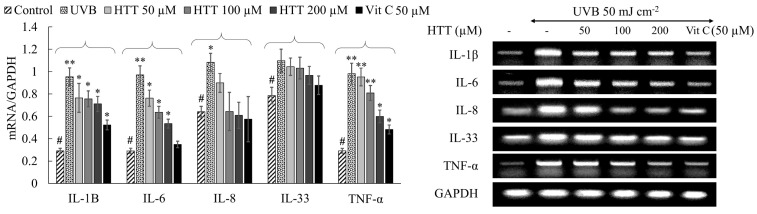
Effects of HTT towards the downregulation of inflammatory cytokine transcription in UVB-induced HDFs. Cells were harvested 18h after the UVB exposure and used for the analysis. Results represent the mean ± SD (error bars) of three independent experimental trials (*n* = 3). *p*-values less than 0.05 “*” and 0.001 “**” were considered as significant compared to the control group “#”.

The transcriptional levels of some key inflammatory cytokines were investigated to evaluate the anti-inflammatory potential of HTT in UVB exposed HDFs. It was evident from Figure 4 that UVB exposure would upregulate the levels of inflammatory cytokines IL-1β, 6, 8, 33, and TNF-α as compared to the control. These observations are parallel with the activation of NF-κB and MAPK signaling, which are upstream modulators of inflammatory cytokines. In HTT pretreated HDFs, the cytokine levels dose-dependently reduced indicating its anti-inflammatory potential.

### 2.5. HTT Downregulated UVB-Induced MMP Expression in HDFs

**Figure 5:**
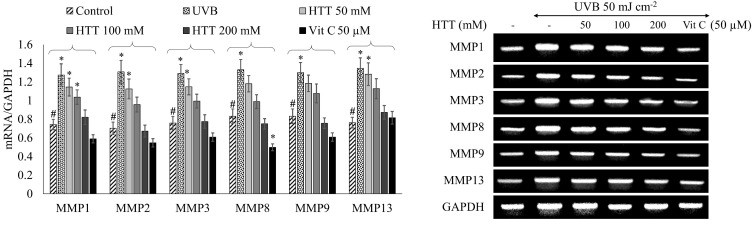
Effects of HTT towards the downregulation of Matrix metalloproteinases (MMPs) in UVB-induced HDFs. Cells were harvested 24 h after the UVB exposure and used for the RT-PCR analysis. Results represent the mean ± SD (error bars) of three independent experimental trials (*n* = 3). *p*-values less than 0.05 “*” were considered as significant compared to the control group “#”.

Matrix metalloproteinases (MMPs) are implicated in UVB-induced photoaging of human skin via the degradation of extracellular matrix (ECM) components [17]. It was shown from RT-PCR analysis (Figure 5) that UVB radiation of 50 mJ cm^−2^ caused a significant upregulation of MMP1, MMP2, MMP3, MMP8, MMP9, and MMP13 in HDFs. Cells pretreated with HTT indicated a dose-dependent downregulation in UVB-induced expression of MMPs. HTT dose 200 µM had profound MMP1, MMP2, MMP3, MMP8, and MMP9 inhibitory effects even surpassing the level of control. These outcomes suggest that HTT has protective effects against UVB-induced ECM degradation.

### 2.6. HTT Reduced Collagenase and Elastase Activity in UVB-Stimulated HDFs

**Figure 6:**
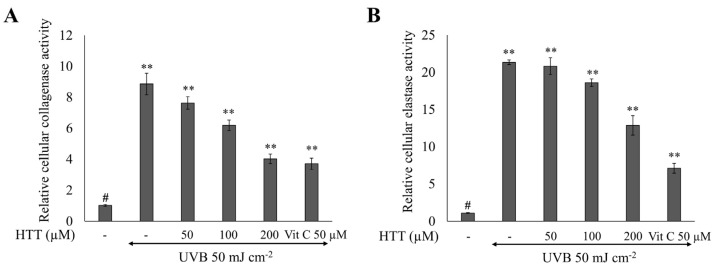
Effects of HTT towards the inhibition of collagenase and elastase activity in UVB-induced HDFs. Effects of HTT on (**A**) collagenase and (**B**) elastase activity in UVB exposed cells. Cells were harvested 24h after the UVB exposure and cell lysates were used for the analysis of collagenase and elastase activity. Results represent the mean ± SD (error bars) of three independent experimental trials (*n* = 3). *p*-values less than 0.001 “**” were considered as significant compared to the control group “#”.

Collagenase and elastase are responsible for the respective degradation of collagen and elastin in the extra-cellular matrix. Inhibition of these enzymes plays a major role in preventing skin wrinkling. The activity of collagenase and elastase was high in UVB stimulated HDF cell lysates compared to the control (Figure 6). Similar observations have been reported by Wang et al. (2020) [3]. Cells pretreated with HTT indicated a reduction of collagenase and elastase activity along with the dose, indicating protective effects of HTT. However, present results deviate from downregulated MMP mRNA expression levels which were profoundly low.

## 3. Discussion

Skin aging is a highly concerning social issue today as its health and attractiveness are regarded as one of the most important indicators of general "well-being" and "health" perception. Though it is a normal biological process, certain internal and external factors including exposure to sunlight would accelerate premature skin aging (photoaging). Overproduction of ROS, caused by UVB exposure leads to lipid peroxidation, which is followed by numerous inflammatory processes, DNA damage, and cell death. This causes clinical and histological changes in the skin such as fine and coarse wrinkling, dryness, mottled pigmentation, and loss of skin tone. Hence, skin-permeable antioxidants that reduce ROS production may help to alleviate skin aging symptoms [6]. 

Natural products have long been acknowledged and inspired as cosmetic and medicinal ingredients due to their robust nature. A growing trend has recently put forward marine algae as rich sources of bioactive natural products. *S. horneri* is among such seaweed that is extensively explored for its bioactive constituents including fucoidans [18], mojabanchromanol [19], and (−)-loliolide [11]. In the last few years, drifting and inundating biomasses of brown seaweed (*S. horneri*), referred to as “golden tides”, has caused a devastating impact on coastal ecosystems and the local economy in parts of South Korea, China, and Japan. Hence, *S. horneri* has recently acquired the center of attention as a sustainable bioresource with potential use as feedstocks for numerous industries. The compound (−)-loliolide, also known as 6-hydroxy-4,4,7a-trimethyl-5,6,7,7a-tetrahydrobenzofuran-2(4H)-one (HTT) is thought to be an oxidation product of zeaxanthin [20]. The present analysis used (−)-loliolide, isolated from the abundant seaweed, *S. horneri.* (−)-Loliolide was previously isolated from numerous plants, algae, as well as animals [20,21,22]. Based on present outcomes, (−)-loliolide indicated antioxidant and cytoprotective effects against UVB exposure in HDFs. Previous studies reveal that HTT possesses antioxidant, anti-inflammatory, wound-healing, hair growth-promoting, and cell senescence inhibiting activities [23,24]. (−)-Loliolide doses effective for ameliorating UVB-induced detrimental effects were established as 50–200 µM. Results of fluorometric analysis for intracellular ROS levels were further validated by fluorescence microscopic and more accurate flow cytometric (FACS) analysis (supplementary materials, Figure S1). 

UVB irradiation produces a post-irradiation-dependent rise in intracellular ROS, as well as changes in cell membrane fluidity and mitochondrial membrane depolarization, both of which have detrimental effects on cells and can lead to apoptosis. Mitochondria are the major ROS producers and targets of ROS in cells. ROS cause deformities in mitochondrial structure altering mitochondrial membrane potential (ΔΨm) with a subsequent discharge of apoptosis-inducing factors [14]. Hence, examining the health status of mitochondria is imperative for understanding the effects of drugs on ROS-mediated apoptosis. The results herein confirmed amelioration of ΔΨm upon HTT treatment. Cells undergoing apoptosis were first identified by monitoring apoptotic bodies indicated by nuclear condensation and fragmentation using the stain Hoechst 33342. It is a DNA-specific fluorescent stain that binds to regions of DNA that are rich with adenine-thymine [25]. The reduced occurrence of apoptotic bodies upon HTT treatment is indicative of its protective effects against UVB exposure. Flow cytometry was used to determine the proportion of cells in different stages of the cell cycle (sub-G_1_, G1, S, G2, and M) following PI labeling. Accumulation of cells in the sub-G_1_ stage of the cell cycle is considered a biomarker of DNA damage, and the appearance of this peak is related to apoptotic body formation [12]. The population of sub-G_1_ cells gradually reduced with the dose-dependent HTT treatment suggesting a reduction of apoptosis. Herein, the outcomes of restoring mitochondrial health status and apoptosis are in agreement.

Oxidative stress is a key process that drives mitochondria-mediated apoptosis. The permeabilization of the mitochondrial outer membrane initiates mitochondrial apoptosis by releasing apoptogenic proteins [26]. Cytochrome c is one of the apoptogenic proteins, that forms an apoptosome by associating with apoptosis protease activating factor (APAF-1) and pro-caspase 9. This complex increases caspase 9 activation, which in turn activates effector caspases, jointly coordinating the execution of apoptosis. The Bcl-2 family of proteins are involved in mediating the permeability of the mitochondrial membrane. They include anti-apoptotic proteins, Bcl-2 and Bcl-xL, and pro-apoptotic proteins, Bax, Bak, Bok, Bad, Bik, Bid, Puma, Bim, Bmf, and Noxa. Their functions are interrelated in an opposing manner. Multi-domain Bcl-2 family members generate channels in the outer mitochondrial membrane causing the release of apoptogenic proteins from the intermembrane gap. The mitochondrial apoptosis pathway is equivocally mediated by p53 which gets activated upon pro-apoptotic stimuli. Post-translational modifications of activated p53 drive the production of APAF-1 and numerous pro-apoptotic proteins of the Bcl-2 family. Moreover, p53 activates transcriptionally independent death pathways. The outcomes of the present analysis favor execution of apoptosis in UVB exposed cells while inhibition was observed with dose-dependent treatment of HTT. 

MMP expression can be promoted by UVB-induced activation of upstream transcription factors such as activator protein-1 and NF-B p65. The MAPK signaling pathway, on the other hand, controls the activation of activator protein-1 and, as a result, IκB kinase, phosphoinositide 3-kinase-Akt, and p38 MAPK. Above mediators can sequentially activate NF-κB prompting the production of inflammatory cytokines other than MMPs [27]. Thus, inhibiting UVB-induced activation of NF-κB and MAPK pathway proteins is critical for ameliorating skin photoaging. The outcomes of the present analysis indicated that the treatment of HTT dose-dependently inhibited activation of molecular mediators responsible for nuclear translocation of activator protein-1 and NF-κB p65 transcription factors. Immunofluorescence analysis of NF-κB p65 nuclear translocation further confirmed the UVB protective potency of HTT.

According to histopathological studies, connective tissue composed primarily of collagen, elastin, fibronectin, and proteoglycans confronts major alterations of photodamaged skin. As collagen and elastin fibrils are responsible for maintaining the strength and elasticity of the skin, their degeneration causes the skin to become wrinkled and less youthful in appearance. The connective tissue degradation is caused by increased MMP activity [9]. Prototypical collagenases in humans are MMP1, MMP8, and MMP13. MMP-1, -8, and -13 can readily cleave interstitial collagens. Additionally, MMP2 and MMP14 are capable of cleaving collagen. The principal function of MMP2 and MMP9 would be the digestion of gelatin [28]. In UVB exposed skin, MMP2 and MMP9 cause the degradation of the epidermal basement membrane that controls epidermal differentiation and adhesion between epidermis and dermis [29]. Hence, inhibiting unregulated MMP synthesis would protect the skin from UVB-induced aging symptoms. Analysis of MMP transcription by RT-PCR indicated that the prompt increase of MMP1, MMP2, MMP3, MMP8, MMP9, and MMP13 productions in HDFs upon UVB exposure is downregulated by HTT treatment. These findings are consistent with the dose-dependent suppression of transcription factor NF-B p65 nuclear translocation and its upstream mediators. 

Collagenase and elastase activity in HDF lysates were evaluated in support of MMP transcription analysis. The significant increase of the relative collagenase and elastase activity in UVB stimulated cells relate to the observed increase of MMP1, MMP2, MMP3, MMP8, MMP9, and MMP13. These findings were consistent with prior research [3]. HTT treatment dose-dependently reduced the collagenase and elastase activity. When compared, HTT was more effective in inhibiting collagenase, than elastase. However, considering the dose-dependent downregulation of MMPs, inhibition of NF-κB p65 and its upstream mediators, the inhibition of collagenase and elastase activity did not reach a level nearing that of the control at the 200 µM HTT dose. This could be due to the differences in time points of transcription (MMPs) and translation (collagenase and elastase) that depend on the cell harvesting time, which was 24 h after UVB exposure. 

The expression of MMPs in HDFs is regulated further by pro-inflammatory cytokines such as IL-1β, IL-6, IL-8, IL-33, and TNF-α [30]. Hence, their levels were investigated in UVB-irradiated HDFs primed with and without different concentrations of HTT. UVB exposure upregulated the expression of pro-inflammatory cytokines, IL-1β, IL-6, IL-8, IL-33, and TNF-α. HTT treatment had a preventive potential for downregulating expression levels of IL-1β, IL-6, IL-8, IL-33, and TNF-α in UVB exposed HDFs. The downregulation of pro-inflammatory markers may be due to the inhibition of transcription factor NF-κB p65 and its upstream mediators. Collectively present analysis suggests that HTT possesses protective effects against UVB exposure-induced skin aging.

The effectivity of a drug candidate for topical application mainly depends on the skin permeability. As numerous constraints limit the experimental evaluation of skin permeability, it is initially evaluated by computational tools. Herein, the skin permeation coefficient (Kp) of HTT in the stratum corneum was analyzed utilizing the Kp calculation available from the SwissADME free web tool (http://www.swissadme.ch/index.php, accessed on 20 April 2021) [31], which was initially developed based on the QSAR model developed by Potts and Guy [32]. The skin permeability of a drug is mainly determined by molecular size and lipophilicity. According to Figure S2 (supplementary materials), HTT indicated a Log Kp value of −6.79 cm/s indicating that it is desirable to employ in topical applications. According to the model by Potts and Guy, the lowest estimated permeability was reported for clindamycin (Log Kp = −9.6) while the highest predicted permeability values are reported for lipophilic compounds such as bexarotene (Log Kp = −4.5), tazarotene (Log Kp = −4.7) and adapalene (Log Kp = −5.3) [32].

The use of natural compounds is an attractive strategy for developing cosmetic formulations with antioxidant and photoprotective properties. Based on this research work, the outcomes suggest a satisfactory intracellular antioxidant activity for HTT against UVB-induced oxidative stress, with possibilities to use as a bioactive agent in a sunscreen formulation. Among UV radiation emitted from the sunlight, UVA is considered more harmful and abundant compared to UVB and can penetrate deeper into skin layers [2]. Considering antioxidant effects of HHT which ameliorated damaging effects of UVB and implicated oxidative stress, it may provide effective protection against UVA radiation. Further studies are needed to investigate the effects of UVA and UVB radiation on different skin cells such as keratinocytes, melanocytes, and fibroblasts. According to recent literature, basic research for skin aging is moving beyond the use of monolayer single-cell models to organotypic skin culture models and human skin explants. These multi-component and complex three-dimensional structures of cutaneous biopsies could provide reliable outcomes as they collaborate various factors such as the spatial association between different types of skin cell layers and matrix effects [33]. Hence, further studies regarding the UV protective effects of HTT could be conducted using the aforementioned multi-layer skin culture models. In UV-exposed skin, the pro-inflammatory cytokines are released by numerous cells including keratinocytes, fibroblasts, and melanocytes. These cytokines induce inflammatory responses in cells located within their microenvironment. Overproduction of inflammatory cytokines causes infiltration and activation of neutrophils causing degranulation, with subsequent MMP production, resulting in photoaging. Therefore, further studies are required to assess the UVB protective effects of HTT in different cell types integrated with the UVB exposed murine model.

## 4. Materials and Methods

### 4.1. Materials

Human dermal fibroblasts (HDF; ATCC PCS-201-012) were purchased from the Korean Cell Line Bank (KCLB, Seoul, Korea). Ham’s F-12 nutrient mix, Dulbecco’s Modified Eagle Medium (DMEM), Fetal bovine serum (FBS), and the mixture of penicillin-streptomycin were obtained from GIBCO INC., NY, USA. Azo dye-impregnated collagen, n-succinyl-Ala-Ala-Ala-p-nitroanilide, phenylmethylsulfonyl fluoride, (AAAPN)3-(4,5-dimethylthiazol-2-yl)-2,5-diphenyltetrazolium bromide (MTT), Triton™ X-100, Hoechst 33342, propidium iodide (PI), 2′ 7′-dichlorodihydrofluorescein diacetate (DCF-DA), paraformaldehyde, Trizol, chloroform, and isopropanol were purchased from Sigma-Aldrich Co (St. Louis, MO, USA). SuperSignal™ West Pico PLUS and DEPC water was obtained from Thermo Fisher Scientific (Rockford, IL, USA). An Ace-α-^®^ cDNA synthesis kit was obtained from ReverTra (Osaka, Japan). Primary and secondary antibodies, including Prolong^®^ Gold AntiFade Reagent with DAPI, goat serum, and DyLight™ 554 Phalloidin, were purchased from Cell Signaling Technology (Beverly, MA, USA). 

The (−)-loliolide isolation procedure from *Sargassum horneri* is described in our previous publication Kim *et al*. 2020 [11]. Briefly dried powder of *S. horneri* was extracted using 70% ethanol with sonication and the crude (SHE) was obtained by rotary evaporation. SHE was dispersed in water and partitioned into chloroform. The chloroform fraction after evaporating the solvent was subjected to further fractionation by high-performance centrifugal partition chromatography (HPCPC) using a two-phase solvent system comprised of 5:5:5:5 (*v/v*) *n*-Hexane, ethyl acetate, methanol, and water. Further purification and isolation were carried out by a Waters high-performance liquid chromatography (Milford, MA, USA) using a C18 SunFire column (4.6 × 150 mm) and using a gradient solvent system of acetonitrile and water. The structure was confirmed by NMR (^1^H and ^12^C) and mass spectroscopy (HPLC-DAD-ESI/MS). 

### 4.2. Cell Culture

HDFs were maintained at 37 °C in a culture medium composed of DMEM, 25% F-12 supplement, 10% FBS, and 1% of antibiotics mix. Cells were sub-cultured once every five days with the media being replaced once every two days. Cells under exponential growth (passage 3–6) were used for the experiments. For dose-response assessment of UVB and HTT, HDFs were seeded at 1 × 10^5^ cells mL^−1^ concentration in well plates. After 24 h, wells were either treated with different concentrations of HTT or exposed to different UVB energies. During UVB irradiation, the culture media was replaced by PBS and afterward incubated with serum-free culture media. MTT assay was conducted after 24 h [34]. A SpectraMax M2 microplate reader (Molecular Devices, San Jose, CA, USA) was employed for measuring the absorbance.

### 4.3. UVB Exposure of HDFs and Measuring Oxidative Stress

HDFs were seeded at 1 × 10^5^ cells mL^−1^ concentration in 24 well plates. After 24 h, wells were treated with optimized HTT concentrations and incubated for 2 h. After, the culture media was gently aspirated, once washed with PBS, and substituted by PBS. Wells of the control group were covered using an aluminum foil and the plate was directly placed under the UV light of the Trans Linker CL-1000™ (UVP, Upland, CA, USA). The cells were exposed with 50 mJ cm^−2^ of UVB under the 280–320 nm range. The PBS was swiftly aspirated after the UVB exposure, and the wells were once washed and substituted with serum-free culture media and respective sample doses. The cells were then incubated for 1 h and 24 h, respectively, to assess intracellular ROS levels and cell viability using DCF DA and MTT assays. [34]. SpectraMax M2 microplate reader was employed for fluorometric analysis. Other than fluorometry, DCF DA treated cells were analyzed by a Thermo Fisher Scientific EVOS FL Auto 2 Imaging fluorescence microscope (Rockford, IL, USA), and a CytoFLEX, flow cytometer (Beckman Coulter, Brea, CA, USA).

### 4.4. Evaluating Mitochondrial Depolarization by JC-1 Assay

The HDF treatment and UVB exposure method is similar to the above. Cells were harvested 6 h after the UVB exposure. The assay was conducted using a MitoProbe™ JC-1 assay kit (Thermo Fisher Scientific, Rockford, IL, USA) following manufacturer instructions. Results were analyzed by a CytoFLEX, flow cytometer.

### 4.5. Evaluating Apoptosis

The formation of apoptotic bodies 24 h after the UVB exposure was examined following Hoechst 33342 treatment and by cell cycle analysis following PI staining. Analysis methods are described in our previous publication [25]. The results were analyzed by a CytoFLEX, flow cytometer.

### 4.6. Western Blot Analysis

For evaluating the levels of MAPK and NF-κB molecular mediators, HDFs were harvested after a 1 h incubation period following the UVB exposure. Cytosolic and nuclear cell lysates were prepared by using a Thermo Fisher Scientific NE-PER^®^ Extraction Kit following manufacturer instructions. A western blot analysis was carried out following the previously described method [35].

### 4.7. RT PCR Analysis

Total RNA was isolated using TRI Reagent^®^ (Molecular Research Center, Inc., Cincinnati, OH, USA) and normalized using nuclease-free water. A ReverTra Ace-α-^®^ kit (Toyobo, Osaka, Japan) was used for cDNA synthesis following manufacturer instructions. cDNA amplification was carried out by 30 PCR cycles. The used primer sequences are given in Table 1. The product of RT-PCR was loaded to 1% agarose gels containing 0.5 µg mL^−1^ ethidium bromide and electrophoresed for 20 min at 100 V. The gels were visualized using a WUV-L20 UV transilluminator (witeg Labortechnik GmbH, Wertheim, Germany) [35].

### 4.8. Immunofluorescence Analysis

The protocol of immunofluorescence analysis is described in [34]. In brief, HDFs were seeded in eight well chamber slides. Following sample treatment and UVB exposure, HDFs were incubated for 40 min. Afterward, the wells were gently washed with PBS and left for fixation in 4% paraformaldehyde. After PBS washing, a blocking buffer containing 5% normal goat serum and 0.3% Triton X-100 was added and left for 1 h in PBS. Next, the wells were sequentially incubated with an anti-NF-κB p65 antibody, PBS washed, and incubated in Alexa Fluor^®^ 488 conjugated Anti-Mouse IgG. Following PBS washing, chamber detachment slides were mounted in DAPI containing Prolong^®^ Gold antifade reagent and cover-slipped. The cells were visualized using an EVOS FL Auto 2 Imaging microscope.

### 4.9. Analysis of Collagenase and Elastase Activity in Cell Lysates

Collagenase and elastase activities of the cell lysates were determined according to the protocol of Suganuma et al. (2010) [36]. Briefly, cells were trypsinated and harvested after 24 h from UVB exposure. Cells were lysed using 0.1 M Tris–HCl buffer (pH 7.6) containing 0.1% Triton-X 100 and 1 mM PMSF by 3s sonication for 3s intervals for 10 min in ice (<4 °C). A Pierce BCA protein assay kit was used to measure the protein content in the lysates.

### 4.10. Statistical Analysis

The data are given as mean ± standard deviation of three independent determinations (*n* = 3). The student’s t-test was used to assess the significant differences between the means of the parameters using IBM SPSS Statistics software (Version 24, IBM, Endicott, NY, USA). *p*-values less than 0.05 “*” and 0.001 “**” were considered significant.

## 5. Conclusions

The present study evaluated the protective effects of HTT against UVB-induced detrimental cellular responses on HDFs. HTT suppressed intracellular ROS production in UVB exposed HDFs while increasing cell viability. Amelioration of mitochondrial hyperpolarization and apoptotic body formation was further observed. HTT dose-dependently suppressed NF-κB and MAPK mediated inflammatory responses and expression of pro-inflammatory cytokines, IL-1β, 6, 8, 33, and TNF-α. Moreover, HTT downregulated the UVB-induced expression of MMP1, MMP2, MMP3, MMP8, MMP9, and MMP13 indicating profound anti-wrinkling activity that was further attributable to the inhibition of collagenase and elastase activity. Collectively present evidence implies that HTT possesses protective effects against premature skin aging caused by UVB exposure of HDFs via the suppressing of ECM degradation. The present results have encouraged further studies, which take the effectivity of HTT into account in the UVB-exposed murine model.

## Figures and Tables

**Table 1:** Forward and reverse primer sequences for PCR analysis.

Target Gene		Primer Sequence (5′ to 3′ Direction)
**IL-1β**	Forward	TGT CCT GCG TGT TGA AAG ATG A
	Reverse	CAG GCA GTT GGG CAT TGG TG
**IL-6**	Forward	GAT GGC TGA AAA AGA TGG ATG C
	Reverse	TGG TTG GGT CAG GGG TGG TT
**IL-8**	Forward	ACA CTG CGC CAA CAC AGA AAT TA
	Reverse	CAG GCA GTT GGG CAT TGG TG
**IL-33**	Forward	GAT GAG ATG TCT CGG CTG CTT G
	Reverse	AGC CGT TAC GGA TAT GGT GGT C
**TNF-α**	Forward	GGC AGT CAG ATC ATC TTC TCG AA
	Reverse	GAA GGC CTA AGG TCC ACT TGT GT
**MMP1**	Forward	CTGAAGGTGATGAAGCAGCC
	Reverse	AGTCCAAGAGAATGGCCGAG
**MMP2**	Forward	GCGACAAGAAGTATGGCTTC
	Reverse	TGCCAAGGTCAATGTCAGGA
**MMP3**	Forward	CTCACAGACCTGACTCGGTT
	Reverse	CACGCCTGAAGGAAGAGATG
**MMP8**	Forward	ATGGACCAACACCTCCGCAA
	Reverse	GTCAATTGCTTGGACGCTGC
**MMP9**	Forward	CGCAGACATCGTCATCCAGT
	Reverse	GGATTGGCCTTGGAAGATGA
**MMP13**	Forward	CTATGGTCCAGGAGATGAAG
	Reverse	AGAGTCTTGCCTGTATCCTC
**GAPDH**	Forward	CGT CTA GAA AAA CCT GCC AA
	Reverse	TGA AGT CAA AGG AGA CCA CC-

## Data Availability

The data presented in this study are available on request from the corresponding author.

## Supplementary Materials

The following are available online at https://www.mdpi.com/article/10.3390/md19080435/s1, Figure S1: Effects of HTT in reducing UVB-induced intracellular ROS levels, Figure S2 Predicted physicochemical descriptors, ADME parameters and pharmacokinetic properties of HTT to act as a drug.
