# Supplementary material for: (−)-Loliolide Isolated from Sargassum horneri Abate UVB-Induced Oxidative Damage in Human Dermal Fibroblasts and Subside ECM Degradation
Source: Mar Drugs. 2021 Jul 30;19(8):435. doi: 10.3390/md19080435 (PMC8399698; doi:10.3390/md19080435)
Supplement: Supplementary file 1 [file marinedrugs-19-00435-s001.zip › marinedrugs-1295960-supplementary.pdf]

marine drugs

Supplementary materials

Marine drugs

Special issue - Marine Products with Anti-allergic and Anti-inflammatory Activities

(−)-Loliolide isolated from Sargassum horneri abate UVB-induced oxidative damage in human dermal fibroblasts and subside ECM degradation

Ilekuttige Priyan Shanura Fernando [1], Soo-Jin Heo [2], Mawalle Kankanamge Hasitha Madhawa Dias [3], Disanayake Mudiyanselage Dinesh Madusanka [3], Eui Jeong Han [3], Min Ju Kim [3], K.K.Asanka Sanjeewa [4], Kyounghoon Lee [5,6,]* and Ginnae Ahn [1,3,]*

HTT attenuated intracellular ROS level in UVB exposed HDFs

Intracellular ROS levels of HDFs were analyzed by fluorescence microscopy and flow cytometry. Per Figure S1A and S1B, UVB exposure caused a significant increase in intracellular ROS levels indicated by increased green fluorescence intensity compared to the control. HTT treated HDFs indicated a dose dependent reduction of the fluorescence intensity suggesting its ROS inhibitory effects.

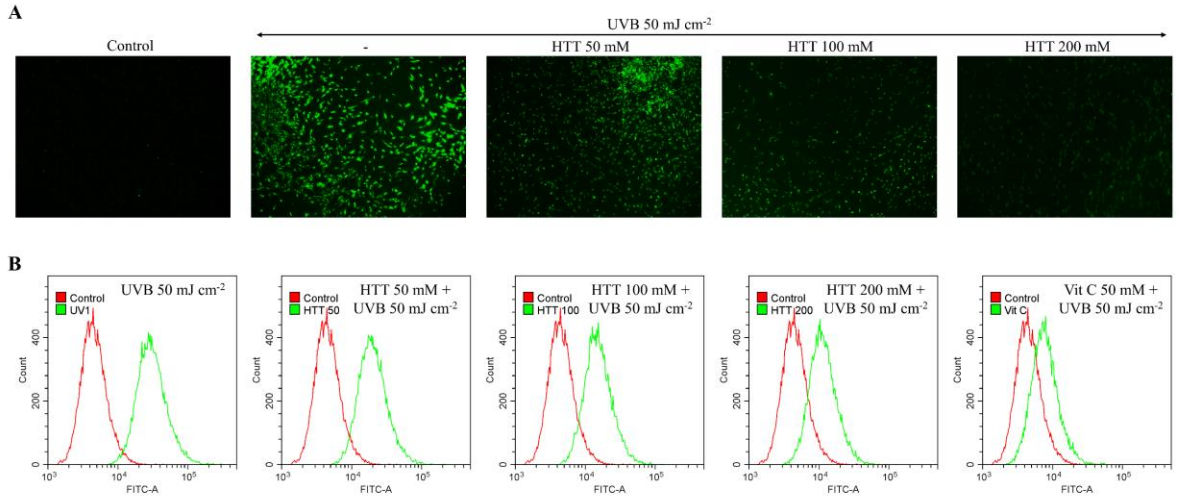

Figure S1. Effects of HTT in reducing UVB-induced intracellular ROS levels. Evaluation of ROS levels by (A) fluorescence microscopy and (B) flow cytometry. Intracellular ROS level was measured 2h after the stimulation. The cells were stained with 2′ 7′-dichlorodihydrofluorescein diacetate (DCF-DA). Results represent the mean ± SD (error bars) of three independent experimental trials (n=3). Bars with different letters are significantly different at P < 0.05.

marine drugs

Predicting skin permeability of HTT

The effectivity of a drug candidate depends on numerous pharmacokinetic parameters including the stability and bioavailability of the drug at the target site, for a sufficient duration. Herein the skin permeability of HTT should be substantial to employ it as a UVB protective substance. The skin permeability of an exposed drug is generally evaluated by determining the skin permeation coefficient (Kp) of the drug in the stratum corneum. However, numerous constraints limit the experimental evaluation of Kp value. The analysis utilizes the Kp calculation available from SwissADME free web tool (http://www.swissadme.ch/index.php) [1], initially developed based on QSAR model developed by Potts and Guy [2]. The skin permeability of a drug is mainly determined by molecular size and lipophilicity. HTT indicated a Log Kp value of -6.79 cm/s indicating that it is desirable to employ in topical applications. According to the model by Potts and Guy, the lowest estimated permeability was reported for clindamycin (Log Kp = -9.6) while the highest predicted permeability values are reported for lipophilic compounds such as bexarotene (Log Kp = -4.5), tazarotene (Log Kp = -4.7) and adapalene (Log Kp = -5.3) [2].

Molecule 1

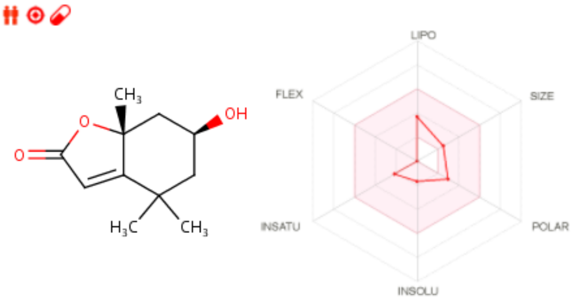

SMILES O[C@@H]1C[C@@]2(C)OC(=O)C=C2C(C1)(C)C

Physicochemical Properties	
Formula	C11H16O3
Molecular weight	196.24 g/mol
Num. heavy atoms	14
Num. arom. heavy atoms	0
Fraction Csp3	0.73
Num. rotatable bonds	0
Num. H-bond acceptors	3
Num. H-bond donors	1
Molar Refractivity	52.51
TPSA	46.53 Å²

Lipophilicity	
Log $P_{o/w}$ (iLOGP)	2.01
Log $P_{o/w}$ (XLOGP3)	1.00
Log $P_{o/w}$ (WLOGP)	1.41
Log $P_{o/w}$ (MLOGP)	1.49
Log $P_{o/w}$ (SILICOS-IT)	1.86
Consensus Log $P_{o/w}$	1.55

Water Solubility	
Log *S* (ESOL)	-1.69
Solubility	4.04e+00 mg/ml ; 2.06e-02 mol/l
Class	Very soluble
Log *S* (Ali)	-1.57
Solubility	5.33e+00 mg/ml ; 2.72e-02 mol/l
Class	Very soluble
Log *S* (SILICOS-IT)	-1.82
Solubility	3.00e+00 mg/ml ; 1.53e-02 mol/l
Class	Soluble

Pharmacokinetics	
GI absorption	High
BBB permeant	Yes
P-gp substrate	No
CYP1A2 inhibitor	No
CYP2C19 inhibitor	No
CYP2C9 inhibitor	No
CYP2D6 inhibitor	No
CYP3A4 inhibitor	No
Log K_p (skin permeation)	-6.79 cm/s

Druglikeness	
Lipinski	Yes; 0 violation
Ghose	Yes
Veber	Yes
Egan	Yes
Muegge	No; 1 violation: MW<200
Bioavailability Score	0.55

Medicinal Chemistry	
PAINS	0 alert
Brenk	0 alert
Leadlikeness	No; 1 violation: MW<250
Synthetic accessibility	3.63

Figure S2. Predicted physicochemical descriptors, ADME parameters and pharmacokinetic properties of HTT to act as a drug. The calculation was carried out by submitting the SMILES specifications of HTT to SwissADME free web tool (http://www.swissadme.ch/index.php).

References

1. Hay, M.; Thomas, D. W.; Craighead, J. L.; Economides, C.; Rosenthal, J., Clinical development success rates for investigational drugs. *Nature Biotechnol.* **2014,** 32.
2. Potts, R. O.; Guy, R. H., Predicting skin permeability. *Pharm. Res.* **1992,** 9, (5), 663-669.
